# Supplementary material for: Are carbon emissions trading and green financial instruments synergistic? -Comprehensive quantitative research based on content analysis
Source: PLoS One. 2024 Mar 7;19(3):e0298601. doi: 10.1371/journal.pone.0298601 (PMC10919601; doi:10.1371/journal.pone.0298601)
Supplement: S1 Data — (ZIP) [file pone.0298601.s001.zip › Data/地级市Z_F工作报告.zip!/╣π╢1⁄2/├╖╓▌╩╨/└┤╫╘╣1⁄2╓┌║┼í╛┬φ┐╦╩2╛▌═°í┐_2012.docx]

这次全会，是梅州认真贯彻落实省委十届十一次全会和市第六次党代会精神，动员全市上下加快绿色崛起、推进跨越发展，建设富庶美丽幸福新梅州的一次重要会议。今天上午，泽君同志代表市委常委会作了一个很好的报告，在总结去年主要工作的基础上，对今年工作提出了新的要求。泽君同志的报告实事求是把握规律，紧密结合我市发展条件、产业基础、区域环境和资源禀赋，按照科学发展的要求，进一步深化和丰富了实现绿色的经济崛起的思路，明确了实现的路径和载体。报告主题鲜明，目标明确，在对我市当前经济社会发展中的优势与劣势，进行深刻分析的基础上，动员全市党员干部包容开放，改革创新，推动红色土地实现绿色崛起，让大家有一种坐不住、等不起的感觉，切实增强了干事创业的责任感和紧迫感。希望同志们认真学习，深刻领会和贯彻落实好这次全会精神特别是泽君书记的重要讲话精神，只争朝夕，真抓实干，锐意进取，奋发有为，全力把有利于梅州实现绿色的经济崛起的各项事情办实办好。

下面，我就如何贯彻落实好这次全会精神，围绕实施“一园两特带动一精”产业发展战略，实现今年全市经济社会发展主要预期目标，从政府工作的角度，强调抓好十项工作：

1、全力抓好高新技术产业园区建设。按照省“双转移”四年大发展的考核要求，力求园区建设和招商引资有新的突破。一是要加快园区基础建设。畲江片区要切实增强园区服务配套能力，力争新自来水厂、碧桂园四星级酒店 6 月底前建成使用，新梅江大桥、市第一职校园区分校12 月底前竣工，220 千伏变电站提前动工建设。当前，要抢抓春季植树造林的大好时机，做好园区主干道、山头、厂房的美化绿化，提升生态园区水平。东升片区要依法依规清理回收闲置土地和厂房，优化园区企业，美化园区环境。蕉华园区要加快水电路等基础设施建设力度，规划建设休闲养生谷和影视基地。丰顺、兴宁、五华及其他各县也要结合各自的产业实际，抓好工业园区建设，推动工业项目向园区集聚发展。今年，要重点推进丰顺埔寨空港高科技与物流产业园区建设，争取一年见成效、三年上规模、五年大发展。二是要力促项目落地建设。一方面，要加快进园项目建设。当前重点要推进南方稀土科技城、广汽（梅州）汽车零部件及五羊摩托产业园、远望新能源产业园等“园中园”建设，力促赛翡蓝宝石、紫晶光电等投产项目早日达产见效益，五羊摩托、珠江啤酒等在建项目早日建成投产，力争今年园区产值和税收均增长50%以上。另一方面，要努力引进大项目、好项目。要积极争取乡贤回乡投资兴业。突出抓好产业招商，主要领导要主动出击，瞄准大型企业和行业龙头企业，亲自洽谈、全程跟踪，力争取得好的成效。要认真落实好招商引资考核办法，使干得好的有劲头、干得差的有压力。同时，要积极鼓励梅州卷烟厂、塔牌集团、宝丽华等本地骨干企业增资扩产，技改升级，做大做强支柱产业。三是要千方百计破解园区发展难题。当前，我市园区建设面临用地难、融资难、用工难等问题。市高新区、国土等部门要密切配合，盘活存量，拓展增量，依法依规加大征地拆迁力度，确保能按计划满足引进企业的用地需求。市财政、金融等部门要争取上级支持，加强与金融机构合作，探索建立投融资服务平台，拓宽企业融资渠道，为园企建设和进园企业缓解资金困难。市人社、教育、经信、招商等部门要加强协调，建立有效的联动机制，大力推进校企合作、校园（工业园）合作，解决进园企业用工难问题。同时，要积极跟进园区在建项目的服务工作，及时解决企业建设过程中出现的各种困难和问题。

2、全面推进广东梅州文化旅游特色区建设。抓好广东梅州文化旅游特色区建设，是当前的一项重要工作任务。一是要抓紧完善规划。要按照特色区的发展定位，尽快完成特色区总体规划，特别是要重点抓好雁洋核心区、梅江区、丰顺三大区域的控制性详细规划，以高水平规划引领高起点建设。二是要抓出一批亮点。一方面，要加快推进特色区道路、水利、生态等基础设施建设，尽快规划新建一批游客驿站、休闲走廊等游客服务设施。另一方面，要动工建设一批特色区重点项目。梅县要力争上半年动工建设“叶帅故园·客家天堂”、“桥溪古韵·世外桃源”、三乡村野公园三个重点项目；梅江区上半年要拿出攀桂坊、红杏坊等古街老巷的保护开发实施方案，下半年要突破 2-3 个改造开发项目；丰顺县要尽快拿出温泉资源整合方案，加快规划建设客潮温泉城。相关县（区）要按照特色区总体规划要求，争取上半年谈成 2-3 个项目，到年底都有 1 个以上项目建成。三是要提高知名度和影响力。市旅游、招商等部门要加强宣传推介，认真编印好特色区的招商推介手册，精心策划好主题突出的市场营销活动。要切实加强与中央电视台、凤凰卫视、中国旅游报、南方日报等媒体及有影响力的门户网站的战略合作，借助高端媒体进行全方位、立体式宣传。要认真办好客家山歌文化节和各县（市、区）特色节庆活动，加强粤闽赣三省区域旅游合作和联动发展，提高名气、带旺人气、集聚财气。四是要高效运营。要抓紧制定出台《关于加快广东梅州文化旅游特色区发展的决定》及其配套政策，加紧建立完善运营机制和工作制度，推动特色区建设走上快车道。

3、大力推进特色宜居城乡建设。要把加快特色宜居城乡建设作为提升发展竞争力、增强群众幸福感的重要载体，着力建设有特色、有个性、让人记得住的城市。一是要坚持规划引领。围绕“世界客都、宜居家园”发展定位，确保上半年完成《市域城乡总体发展规划》、《城市总体规划》和各县县城规划修编工作。要尽快出台《进一步加强梅州城区规划建设管理工作的意见》，抓紧完善梅州城区控制性详细规划和新一轮的土地利用规划，严格规划控制和管理。二是要突出抓好重点城区。围绕打造七星伴月的城市发展格局，突出抓好江南新区、江北古城、梅县新城和各县县城等重点城区建设。市城乡规划部门要在 4 月底前完成江南新区总体规划和招投标工作，梅江区要在 3 月底前出台新区征地拆迁工作方案，上半年启动征地拆迁工作。要抓好世界客商会馆、世界客都展馆和海航五星级酒店等重要设施建设，彰显客家文化、展示客都风采。要按照“修旧如旧、新旧协调”的原则，抓紧出台江北古城整体开发方案，加快推动项目建设。梅县新城要拉开发展架构，统筹推进山水城、大新城、总部经济区及周边“四馆一场”建设，努力建设适宜“慢生活”的城市新区。各县（市、区）要突出重点、统筹推进新区开发和“三旧”改造，争取年底前都打造1—2 个城市建设新亮点。三是要建设特色乡村。市规划建设部门要抓紧出台《村庄规划建设管理实施办法》，编印《城乡规划建设管理知识读本》，全面铺开乡村建设规划许可证制度，鼓励农民按照客家新民居标准建设新房。要积极支持职业（技工）学校聘请民间“老工匠”办班招徒，为特色城乡建设培育造就一批能工巧匠。各县（市、区）要充分利用山水人文资源，因地制宜，彰显特色，打造名镇名村，建设特色乡村。四是要优化人居环境。要抓住春季造林时机，深入开展“绿满梅州”大行动，广泛发动全市干部群众、海内外乡贤积极参与植树造林，全面完成 300 公里生态景观林带建设任务。要大抓城乡环境整治，巩固提升创建全国卫生城市成果，改善城乡面貌。

四、加快发展精致高效农业。实现农业提质增效，促进农民增收致富，重点要在抓好精致高效农业上下功夫。一是要建好特色农产品基地。要充分利用梅州山地资源优势，围绕市场取向，突出区域特色，加快建设梅州金柚、嘉应名茶、平远慈橙、兴宁油茶、蕉岭毛竹和名贵树木、丰顺优质红薯、五华优质稻、梅江花卉等生产基地。今年要重点扩大慈橙、嘉应茶等优质农产品种植规模，力争新种慈橙 2 万亩、新建优质茶基地 8000 亩。二是要突出龙头示范带动。要把农业龙头企业、农民专业合作组织作为发展精致高效农业的主力军，大力推动土地由农业龙头企业、种养大户集约经营，惠及千家万户。完善激励机制，评选先进企业，充分发挥龙头企业在种植、养殖、加工、销售等领域的带动作用，力争全市新培育 2 家以上省级重点农业龙头企业，20 家市级农业龙头企业，新组建农民专业合作社 30 家。三是要打响特色品牌。要大力实施品牌农业战略，充分发挥科技对农业品牌的支撑作用，积极推进农产品国家地理标志产品原产地认证，加强梅台农业合作交流，深化与省农科院等科研院校的战略合作，加大对农民在种养、加工、销售等各个环节的培训指导，提高产量、提升品质、打响品牌，努力提升农产品的市场竞争力和附加值。四是要抓好市场开拓。要加强农产品整体营销，策划办好各类特色农产品推介会。我们刚刚在广州、香港举办了平远慈橙品尝会、“金柚飘香”招商引资推介会，取得了很好的宣传推介效果。下一步，我们要认真总结这两次活动的成功经验，精心组织好年底在北京举办的梅州金柚、平远慈橙等展销活动，积极拓展中高端市场。

5、突出抓好交通、水利基础设施建设。要继续加大基础设施建设力度，优化梅州发展环境。

一方面，要切实抓好交通重点项目建设。要全力跟进梅州至惠州高铁项目，力争今年完成项目前期工作；要积极做好济广、畲华、梅大高速及其东延线的基础性工作，确保梅大高速及其东延线顺利推进，济广、畲华高速公路年内动工；梅揭高速公路加铺沥青工程年底前完成；争取立项建设浦梅、鹰梅快速铁路；积极谋划五华至丰顺、平远至蕉岭、梅州城区直达揭阳潮汕国际机场等重要交通干线建设，完成 500 公里农村公路硬底化建设；加快实施梅县机场候机楼改建工程。另一方面，要突破一批水利重点建设项目。要大力推进今年计划投资28 亿元的 324 宗重点民生水利工程建设，力争高陂、梅南水利枢纽工程年内立项，全面启动黄塘河、程江河等中小河流域整治工程，改造提升梅州大堤南堤、西堤，切实加强病险水库除险加固、小流域治理、小型农田水利和山洪灾害防治等工程建设。

六、努力保障和改善民生。要围绕就业、就读、就医、社会保障和社会建设，努力做好民生改善的各项重点工作。一是要全力打好扶贫开发“双到”攻坚战。要严格按照省的考核标准，以落实好扶贫项目、加大资金投入为抓手，突出产业帮扶、学技就业帮扶，把扶贫开发“双到”与“双转移”紧密结合，大力开展农村劳动力培训转移就业，确保高质量完成省下达的扶贫开发“双到”任务。二是要提高社会保障水平。要围绕“人人享有社会保障”的目标，建立健全城乡一体化医疗保险制度、社会保障和基本公共服务体系，确保参保人数和基金征收增长10%以上，年底前让 60 周岁以上城乡居民全部享受养老保险待遇。今年要让小学生、60 岁以上老人、残疾人等群体免费乘坐梅城公交车。市县两级要着力办好人民医院和妇幼保健院、中医院，大力推进梅州市田家炳医院心脑血管诊疗中心改造扩建。要继续抓好农房统保和农村危房改造，全面完成省下达我市 1510 套城镇保障性安居工程建设任务。三是要确保社会稳定和谐。要深入开展“长安杯”创建和“三打两建”行动，强化社会治安综合治理，净化社会环境，规范市场秩序。要强化安全生产“一岗双责”，加强非煤矿山、危化品、烟花爆竹、建筑施工、道路交通等重点行业和重点领域的安全专项整治。要推进市县污水处理厂管网配套建设，开展清洁生产，加大节能降耗，确保主要污染物排放总量控制在省下达的目标内。要完善调解机制和县镇村三级法律援助服务网络，形成三级联动的一体化工作格局。要提高各类应急预案的实用性和针对性，增强处置各类突发事项的能力。要高度重视办好今年的十件民生实事，把好事办实，实事办好。

7、积极创建教育强市。围绕明年实现创建教育强市的目标，努力促进城乡教育均衡发展，确保今年新增 1 个教育强县、10 个教育强镇。市县教育部门要整体谋划，制订实施方案，重点推进梅州城区和各县县城一所中学、一所小学、一所幼儿园的规划建设，大力开展“教育基金百万行”活动，动员全市干部群众和海内外乡贤积极捐资办学。要以东山中学为龙头，以10 所示范性高中为重点，着力打造一批全省甚至全国有影响、有名气的示范学校。要抓紧出台提高职业教育发展水平的实施意见，办好重点职业（技工）学校。要加强教师队伍建设，提升教学质量，认真抓好名教师、名校长评选活动，打响“嘉应名师”品牌。

八、着力加强财税金融工作。要充分发挥财政杠杆作用，引导金融机构服务地方经济发展。

一是要认真做好开源节流工作。要着力培植财源，规范非税收入管理，强化税费征管，借助全省推进基本公共服务均等化，积极研究、主动作为，争取中央和省的更大支持。要严格控制一般性支出，特别是行政管理经费支出，确保有限的财政资金用于刀刃上。二是要大力推进广东农村金融改革创新综合试验区建设。要深化农村金融改革创新，大力完善农村信用体系，加快组建新型农村金融机构，力争今年成功组建 1 家农村商业银行，新增 1-2 家村镇银行，实现乡村金融服务站全市 2040 个行政村全覆盖。三是要加大力度推动企业上市。要把推动企业上市作为最有效的招商引资，加大服务扶持力度，大力培育上市后备企业，重点推动条件较为成熟的企业上市，力争今年新增 3 家上市企业。四是要深化政银企合作。要加强与金融机构的合作，争取信贷规模。要通过举办融资推介会、项目洽淡会等方式，搭建银企合作平台，加强银行与企业对接，对诚实守信、发展前景好的企业加大资金投放，支持企业做强做大。同时，要深入开展信用建设，创新金融服务，防范金融风险，优化金融生态环境。

九、深化体制机制改革。要加大重点领域和关键环节的改革力度，进一步完善推动科学发展的机制体制。一是要深化行政体制改革。要继续深入推进富县强镇事权改革，加大政府职能转移管理力度，年底前编制出台市级第一批政府转移职能目录和购买服务目录。二是要深化住房保障制度、医疗保障制度等民生领域改革。要创新保障房建设的投入机制、分配管理制度和规划建设机制，加快构建商品房和保障房双轨并行的新型住房制度，市住建部门上半年要制定出台具体实施方案。要健全统筹城乡的基本医疗保险制度，完善基本医疗保险筹资和待遇水平调整制度。加快推进县级公立医院改革。三是要创新社会管理体制。积极探索宗亲会、公益会、行业协会等民间组织参与社会建设管理，要加紧出台《进一步培育发展和规范管理社会组织的实施意见》，设立发展扶持基金，大力培育发展社会组织。同时，要围绕诚信体系建设、市场法治化建设、制度防腐反腐等改革，尽快出台和实施专项改革方案，着力在消除制约市场经济发展中深层次矛盾和问题上取得新的突破。

十、切实提高执行能力。做好今年的各项工作，关键要抓好落实，关键要提高执行力。一是要分解任务，明确责任。要认真按照这次全会的部署和今年市委、市政府的重点工作安排，进一步明确责任领导、责任单位，明确具体项目和年度目标完成时间，制订切实可行的工作计划，加强督促检查，务求各项工作有效推进。二是要转变职能，改进作风。市政府各职能部门要率先开展“全力推动梅州科学发展”大讨论活动，政府职能部门和公务员队伍要率先树立包容开放，勇于创新的理念，敢于为梅州创造更好的发展环境和赢得更好的发展机会，负责任、出实招、干实事。三是要加强培训，提高效能。要切实加强干部队伍培训，努力提高公务员队伍的履职能力，力争今年培训干部 4 万人次。要加强效能政府建设，争取上半年完善“市区共建”行政服务中心运行模式，各县（市）要尽快启动县级行政服务中心建设。要增强服务意识，对重大项目、重点企业，实行贴身对点服务，有事马上办、主动办、限时办、急事急办、特事特办。四是要依法行政，廉洁从政。手中掌握着一定权力的公务员，要时刻警醒自己，切记自己的身份与使命，真正做到廉洁奉公，务实为民。

同志们，今年的工作任务非常繁重，我们一定要在省委、省政府和市委的坚强领导下，抢抓机遇，开拓创新，狠抓落实，抓出成效，干出实绩，为全力加快绿色的经济崛起、建设富庶美丽幸福新梅州作出新的贡献。
